# Candidate genes expression profiling during wilting in chickpea caused by *Fusarium oxysporum* f. sp. *ciceris* race 5

**DOI:** 10.1371/journal.pone.0224212

**Published:** 2019-10-23

**Authors:** Cristina Caballo, Patricia Castro, Juan Gil, Teresa Millan, Josefa Rubio, Jose V. Die

**Affiliations:** 1 Área de Genómica y Biotecnología, IFAPA, Alameda del Obispo, Córdoba, Spain; 2 Department of Genetics - ETSIAM, University of Córdoba, Campus de Rabanales, Córdoba, Spain; Tallinn University of Technology, ESTONIA

## Abstract

Chickpea production may be seriously threatened by Fusarium wilt, a disease caused by the soil-borne fungus *Fusarium oxysporum* f. sp. *ciceris*. *F*. *oxysporum* race 5 is the most important race in the Mediterranean basin. Recently, the region responsible for resistance race 5 has been delimited within a region on chromosome 2 that spans 820 kb. To gain a better understanding of this genomic region, we used a transcriptomic approach based on quantitative real-time PCR to analyze the expression profiles of 22 selected candidate genes. We used a pair of near-isogenic lines (NILs) differing in their sensitivity to Fusarium race 5 (resistant vs susceptible) to monitor the transcriptional changes over a time-course experiment (24, 48, and 72 hours post inoculation, hpi). Qualitative differences occurred during the timing of regulation. A cluster of 12 genes were induced by the resistant NIL at 24 hpi, whereas a second cluster contained 9 genes induced by the susceptible NIL at 48 hpi. Their possible functions in the molecular defence of chickpea is discussed. Our study provides new insight into the molecular defence against Fusarium race 5 and demonstrates that development of NILs is a rich resource to facilitate the detection of candidate genes. The new genes regulated here may be useful against other Fusarium races.

## Introduction

Chickpea (*Cicer arietinum* L.) is the second grain legume cultivated in the world with a total cultivated area of 14.5 million hectares [1]. It is mainly used for human consumption and is an essential constituent of the Mediterranean diet. Chickpea is a good and cheap source of protein and for this reason this crop is cultivated in the five continents.

One of the major biotic stresses limiting chickpea yield is its susceptibility to fungal diseases. Among these, Fusarium wilt caused by *Fusarium oxysporum* f. sp.*ciceris* (Foc) is a soil-borne disease widespread in most of chickpea growing areas. Foc is a saprophytic fungus that may survive in soil or debris up to six years causing big yield losses in years of severe outbreaks of the disease [2]. The fungus penetrates the plant via roots, the germ tube penetrates the epidermal cells of plants and later the hyphae extend to root cortical region and colonizes the xylem vessels, thus, preventing the upward translocation of water and essential solutes, resulting in wilt [3–5]. *F*. *oxysporum* f. sp. *ciceris* is pathogenic only on *Cicer* spp. [6] but can also invade root tissues of other grain legumes such as faba bean (*Vicia faba*), lentil (*Lens culinaris*), pea (*Pisum sativum*) and pigeonpea (*Cajanus cajans*) without causing external symptoms [5].

Eight physiological races of the pathogen (0, 1A, 1B/C, 2, 3, 4, 5 and 6) have been reported to infect chickpea. Fusarium race 5 (Foc5) is one of the main problem in the Mediterranean area. In chickpea, resistance reaction inheritance to different races has been reported to be monogenic or oligogenic depending on the race or source of resistance [7,8]. Several studies reported that resistance to Foc5 is controlled by a single gene located on linkage group 2 (LG2) of the chickpea genetic map [9,10]. This gene is clustered with other genes conferring resistance to races 0, 1, 2, 3 and 4. The STMS (sequence tagged microsatellite site) marker TA59 has been widely reported as the most associated with that cluster, and hence it has being used in marker assisted selection [11,12]. A deeper knowledge of this genomic region could provide new markers useful for breeding purposes as well as a better understanding of resistance mechanisms. The recognition and defense by a host plant to its fungal pathogen and the ability of the pathogen to overcome the plant defences, implies a very complex molecular network. In the plant-pathogen interaction four phases can be highlighted: pathogen perception, penetration, colonization and disease establishment [13]. In the last decade, numerous transcriptome, metabolome and proteome studies have been carried out on chickpea-Foc interaction to unveil the hidden clues behind the defense pathways, but all of them focused on Foc race 1. In general, these studies reported that molecular changes in carbon and nitrogen metabolism, in reactive oxygen species (ROS), in primary metabolites (amino acids and sugars), in lignification and in phytoalexins are related with resistant reactions [13–22]. Nevertheless, as far as we know, there are not molecular studies reporting candidate genes for resistance to Foc5.

In a previous study, the comparison between genetic and physical chickpea maps of segregant plant material and near isogenic lines (NILs) made it possible to narrow down to 820 kb the area of chromosome 2 (Ca2) where the resistance gene for Foc5 is located [23]. NILs provide plant material differing only in a small target region of the genome facilitating the detection of candidate genes underlying phenotypes. In chickpea, NILs have been used to produce fine maps and targeting genomic regions associated with agronomic traits [24–26].

The aim of the present study was to determine differential expression of candidate genes for resistance to Foc5. To achieve that goal, we selected a set of genes located within the region of interest in LG2, and we analyzed their expression profile in a pair of NILs (resistant vs susceptible) at different time points after inoculation with Foc5.

## Materials and methods

### Plant material

In this study we used a pair of NILs—RIP8-94-5 resistant (R) / RIP8-94-11 susceptible (S)—segregant to race 5 that were developed by searching residual heterozygosity in advanced RILs (Recombinant Inbred Lines) derived from the cross ILC3279 x WR315 [11]. Line ILC3279 is a kabuli type from the former Soviet Union maintained by ICARDA (International Center for Agricultural Research in the Dry Area) that is susceptible to Foc5. WR315 is a desi type from central India maintained by ICRISAT (International Crops Research Institute for the Semi-Arid Tropics) resistant to all Foc races [27]. In addition, Cr5-9, a selection from *C*. *reticulatum* (PI489777), susceptible to all Foc races, was included as a control.

### *Fusarium oxysporum* f. sp. *ciceris* race 5 inoculation

Seeds of each NILs were sown in trays (41 x 56 x 12 cm) filled with perlite. Plants were grown in controlled conditions under a temperature regime of 25 and 22°C and 12 h photoperiod under fluorescent light. Filter papers with fungus spores provided by Dr. W. Chen (Washington State University, Pullman, USA) were added at PDB medium (potato dextrose broth, 24 g l^-1^) and grown in a minitron incubator chamber at 25°C and 100 rpm under continuous fluorescent light. The liquid cultures were filtered through cheesecloth to remove the mycelium. The spore suspension was then pelleted by centrifugation at low speed (3,000 rpm) for 3 min. After that, the supernatant was discarded and the concentration of spores was adjusted to 1 x 10^6^ spores ml^-1^. Plants at the three to four node stage were inoculated following the method described by Bhatti [28]. After inoculation, the plants were watered daily and supplied with nutrient solution once a week. Root samples were collected and pooled from at least 4 inoculated and non-inoculated plants at 24, 48 and 72 hours post-inoculation (hpi). Samples were frozen in liquid nitrogen immediately after harvesting and stored at −80°C. Two biological repetitions per time-point were performed. Ten inoculated plants remained non-harvested to verify that the inoculation was successful.

### RNA isolation, cDNA synthesis and quality controls

Total RNA from all samples was isolated using the TRISURE reagent protocol (Bioline). RNA concentration was determined by measuring the optical density using a NanoDrop spectrophotometer. Only the RNA samples with A260/A280 ratio between 1.9 and 2.1 and A260/A230 greater than 2.0 were used in the analysis. To avoid any genomic DNA (gDNA) contamination, ~10μg of RNA extracts were treated with TURBO DNase I (Life Technologies) before to cDNA synthesis. Complementary DNAs was synthesized by priming with oligodT_12–18_ (Life Technologies), using SuperScript III Reverse Transcriptase (Invitrogen) following the instructions of the provider. The cDNAs were diluted to a final volume of 20μl. Next, we tested the presence of genomic DNA (gDNA) contamination in the cDNA samples using a primer pair designed in two different exons of the NAD-dependent malic chickpea sequence XM_004510782 (gDNAF, 5’-GTTGATACCAGCAGCAGCAAC-3’; gDNAR, 5’-TTAGTGCCAAAGACAAAGGGGA-’3’). The primer pair was designed to amplify a product of 555 bp using gDNA as template or 180 bp using cDNA as template. In our tests for gDNA contamination, the 555 bp band was not amplified from any of the samples. To infer the integrity of the total RNA and assess the quality of the reverse transcriptase reaction we used a 3‘:5’ amplification ratio assessment [29]. This assay aimed at measuring the integrity of the NAD-dependent malic sequence (XM_004510782). For this assay, we designed two primer pairs (malic_5’F, 5‘-CGACCGTTGTCTGATTTTGTG-3’; malic_5’R, 5‘-GGCCATTTTCAGAACCCCTAA-3’; and malic_3’F, 5‘-GCTTCGAGCAGCAGTTGAAGA-3’; and malic_3’R, 5‘-CTTTTGACATGTGTGCAAGTT-3’) to amplify two cDNA fragments, one from the 5’ end (81 bp) and one from the 3’ region (80 bp) of the malic gene. The fragments are 1,180 and 460 bp, respectively, from the 3’ end of the cDNA. The 3‘:5’ amplification ratio of the malic cDNA fragments was calculated using the comparative Cq method [30]. The average ratio was 1.18 ± 0.59 (mean, sd). All ratios were < 1.5-fold. Only if ratios were > 4.4-fold would RNA quality be deemed inadequate [31]. Therefore, the cDNAs were judged to be suitable for qPCR analysis.

### Primer design and quality controls

Primer sequences were designed to amplify 19 candidate genes within a genomic region of interest delimited between the markers TA59 and CaGM07922, covering 2 Mb, based on the genetic fine-mapping of Foc5 susceptibility loci [23]. We also included three genes previously reported to be involved in molecular defence against Fusarium wilt obtained from a chickpea transcriptome upon Foc1 infection [32]. All PCR primers were tested for specificity using NCBI’s BLAST software. Primers were designed using the following criteria: Tm of 60 ± 1°C and PCR amplicon lengths of 80–100 bp, yielding primer sequences with lengths of 19–23 nucleotides and GC contents of 40–80%. For predicting the secondary structure of the amplicons, we used MFOLD version 3.4 software with default settings of minimal free energy, 50 mM Na^+^, 3 mM Mg^2+^, and an annealing temperature of 60°C [33]. We chose primers that would yield amplicons with minimal secondary structures and melting temperatures that would not hamper annealing (S1 Fig). Designed primers were synthesized by Integrated DNA Technologies (Leuven, Belgium). Table 1 shows the primer sequence and the overall mean real-time PCR amplification efficiency of each primer pair (E) estimated from the data obtained from the exponential phase of each individual amplification plot and the equation (1+E) = 10^slope^ using LinReg software and the criteria of including three-five fluorescent data points with R2≥0.998 to define a linear regression line [34].

**Table 1:** Primers for RT-qPCR. Chickpea identifiers, annotation NCBI, primer sequences, amplicon size and chromosome position. Primer PCR efficiency and PCR product Tm data represent mean values ± sd. PCR efficiencies (E) calculated according to the equation (1 + E) = 10^slope^.

Chickpea ID NCBI	Annotation	Primer Sequence (5‘-3’)	PCR product size (bp)	Chr position	PCR E	PCR product Tm (°C)
LOC101503802	kinesin-like protein FRA1	F-TGCTTCCATTCCACCGAAGCCTG	82	Ca2: 23082299–23082381	2.02 ± 0.09	75.77 ± 0.25
		R-TTGCTTTTGCATGGCCCGTGGT				
LOC101505941	serine/threonine-protein kinase CTR1	F-CGCCTGAGTGGATGGCTCCAGA	98	Ca2: 23219419–23219517	1.99 ± 0.07	78.06 ± 0.16
		R-GGTCACGAGTTCCCACAGGATCA				
LOC101506693	GATA transcription factor 25-like	F-AGGATTCTGGGCAGGACGACAG	85	Ca2: 23277155–23277240	1.94 ± 0.05	78 ± 0
		R-CGCCGCATCATTGGGGTCGATT				
LOC101507659	E3 ubiquitin-protein ligase UPL1-like	F-AGTGCTGCATCGCCAGTTATCCA	73	Ca2: 23441535–23441608	1.95 ± 0.08	77.88 ± 0.29
		R-GCCGAGCCTTGTCCTCCTTGCT				
LOC101508507	probable rhamnogalacturonate lyase C	F-CCAACGGGGTAGCGTTTGTGGT	108	Ca2: 23463108–23463216	1.96 ± 0.07	77 ± 0.11
		R-GCATCTCCTGGTGGTGCCAATCC				
LOC101509037	XIAP-associated factor 1-like	F-GGTGTCCGACGACGAAGG	80	Ca2: 23476846–23476926	1.98 ± 0.09	77.5 ± 0
		R-ACAGCAATACCGGTGATGGC				
LOC101509359	MADS-box transcription factor 23-like	F-ACGGTTTGTTGAAGAAGGCGAAGG	116	Ca2: 23551043–23551159	1.95 ± 0.06	77.43 ± 0.17
		R-TGATCTCATGCTGGTGCTAGCGAA				
LOC101510206	serine hydroxymethyltransferase, mitochondrial-like	F-GGCTCGAGGGTTGAGAAGGTGT	94	Ca2: 23674613–23674707	1.99 ± 0.07	77.16 ± 0.23
		R-TGCCCCCAGGAACCATAGCAGA				
LOC101510544	26S protease regulatory subunit 8 homolog A-like	F-GCCAGGGAGCATGCACCATCAA	105	Ca2: 23699688–23699793	1.97 ± 0.07	78.52 ± 0.10
		R-GCGCTGCACCTCACTATCACCA				
LOC101511605	CBL-interacting serine/threonine-protein kinase 8	F-TGCTTCGGACAACTTGCGGGAC	98	Ca2: 23829711–23829809	1.97 ± 0.05	76.74 ± 0.23
		R-CCCACACGACCAAACATCCGCT				
LOC101495287	auxin-responsive protein IAA8-like	F-TGAGAGGCCTCCTGGTGTCTGTG	92	Ca2: 23950935–23951027	1.97 ± 0.07	76.71 ± 0.25
		R-CCTGTGCCTTGGTAGCTGGTGC				
LOC101495941	MATE efflux family protein 5-like	F-GGTGGGGTGGCAATAGCAATGGT	109	Ca2: 24038256–24038365	1.94 ± 0.06	74.09 ± 0.21
		R-AGCTTGGTGGGACCCATGATTGT				
LOC101496824	sucrose transport protein SUC4	F-ATCTGGCTTTGCGGCCCAGTTT	88	Ca2: 24044382–24044470	1.93 ± 0.06	79.64 ± 0.14
		R-GTCGACCGAATCGGCTTTGGCA				
LOC101497351	bidirectional sugar transporter N3-like	F-ATGCATGGTTCCCTTCGAGTCCA	89	Ca2: 24085883–24085972	1.96 ± 0.08	76.84 ± 0.25
		R-GCCACAATGCATAGAGGTGCTGC				
LOC101497678	glycerol-3-phosphate dehydrogenase [NAD(+)] 2, chloroplastic	F-AACCGGTCTGTCAGGAACTGGAG	93	Ca2: 24092611–24092704	1.96 ± 0.07	76.7 ± 0
		R-TCGCCTGATCCAAGACGCACAC				
LOC101502928	WRKY transcription factor 55	F-AGCACCATTATCATATCCACCAC	80	Ca2: 24128013–24128093	1.96 ± 0.06	68.87 ± 0.10
		R-ACAATTGGGGAGAAATGGTGGT				
LOC101499218	E3 ubiquitin-protein ligase SHPRH	F-CAACACGTGGTCCTTGTTGAGCC	80	Ca2: 24209104–24209184	1.91 ± 0.07	77.7 ± 0.14
		R-TGTCCAATCCGATGTACGCGACT				
LOC101501552	probable inactive receptor kinase At5g58300	F-GGTGGGTGAAGTCTGTGGTTTCTGA	108	Ca2: 24365119–24365227	1.95 ± 0.10	76.29 ± 0.16
		R-TCTGAAGCATCTGCACCATCTCCTC				
LOC101510320	pathogenesis-related protein 5	F-GCTGCACATTTGATGCAACGGGA	89	Ca2: 24740222–24740311	1.93 ± 0.05	76.88 ± 0.24
		R-TGGAGCTGCACCATTCCCATCAC				
LOC101499873	TMV resistance protein N-like	F-TGACCTTACGTGGCACCGCTAT	126	Scaffold	1.97 ± 0.06	76.74 ± 0.22
		R-GCTGCGGATCATTTGACAACCCA				
LOC101490851	20 kDa chaperonin, chloroplastic-like	F-CCAGGGTCTGTGGATGAGGAAGG	99	Ca7: 3352808 - 3352906	1.96 ± 0.06	74.91 ± 0
		R-GCCCTTGAAGTCATTCCCCGCA				
LOC105852647	MLP-like protein 28	F-TGGTGGAGATATTGATGAGCACT	80	Ca7: 45480380 - 45480459	1.97 ± 0.08	72.19 ± 0.25
		R-CGGAAGCACTACCATCAGCC				

### Real-time qPCR assays

PCR reactions were carried out in a CFX Connect Real-Time System thermal cycler (Bio-Rad, Hercules, CA, USA) iTaq Universal SYBR Green Supermix (Bio-Rad) to monitor dsDNA synthesis. Reactions contained 1.5 μl of the diluted cDNA as a template and 0.2 μM of each primer in a total volume reaction of 10 μl. Master mix was prepared and dispensed into individual wells using electronic Eppendorf Xplorer^®^ multipipettes (Eppendorf AG, Germany). The following standard thermal profile was used for all PCRs: polymerase activation (95°C for 3 min), amplification and quantification cycles repeated 40 times (95°C for 3 s, 60°C for 30 s). The specificity of the primer pairs was checked by melting-curve analysis performed by the PCR machine after 40 amplification cycles (60–95°C) and is shown in S2 Fig. Fluorescence was analyzed using Bio-Rad CFX Manager analysis software v2.1. All amplification plots were analyzed using a baseline threshold of 75 relative fluorescence units (RFU) to obtain Cq (quantification cycle) values for each gene-cDNA combination.

### Reference genes selection and qPCR data analysis

For optimal normalization of data, we evaluated the stable gene expression of four references in our dataset. Two candidates encoding a phosphatase protein (*PP2A*), and pentatricopeptide repeat-containing protein (*PPR*) were chosen based on previous reports [35]. We also tested the expression of a transcription factor initiation IIA (*TFIIA*) which had happened to be one of the most stable genes under a variety of conditions [36]. Finally, we also designed primers to amplify a chickpea sequence (*Ca4g26410*) ortholog to an *Arabidopsi*s gene that had showed high stability values across developmental series in cross-species experiments [37,38]. To evaluate the stability of the reference genes, we used the geNorm algorithm and the coefficient of variation (CV) of normalized relative quantities based on the equations defined by the qBase framework [39,40]. Calculations were performed using the advanced quantification model with efficiency correction, multiple reference genes normalization and use of error propagation rules described by Hellemans [40]. The open-source interface for the statistics software R, RStudio (http://www.rstudio.com/), was used to perform exploratory data analysis and build clusters. To gain insights into the biological roles of genes used, we performed a search of Gene Ontology (GO) categories using the UniProt database [41].

## Results and discussion

In the present study, we compared the expression patterns of 22 potential defence-related genes in response to Foc5 inoculation by a real-time qPCR-based strategy. Over the past two decades, our laboratory has been working towards increasing the genetic variability of the cultivated chickpea species using a combination of inter- and intra-specific crosses that are evaluated for complex traits of agronomic importance, such as resistance to Fusarium wilt. Foc is the major soil-borne fungus affecting chickpeas globally and may cause important yield losses under favourable conditions [8]. Particularly, Foc0 and Foc5 races are present in Mediterranean basin, being Foc5 the most virulent. We have previously reported DNA markers targeting Foc0 and Foc5 resistance through linkage analyses, but those were not highly saturated areas [26,42–47]. Recently, we saturated a region located on Ca2, which is likely implicated in resistance to Foc5. That region is delimited by SNP markers and includes twenty-six genes [23]. In the present study we selected nineteen annotated genes within this genomic region. We also included three genes beyond the target region that have been related with chickpea resistance against Fusarium races 1, 2 and 4 in previous reports [32]. We selected a pair of NILs differing in their sensitivity to Foc5 to monitor the gene expression patterns during a time-course of 24-48-72 hours following the pathogen inoculation.

### Disease development

**Fig 1:**
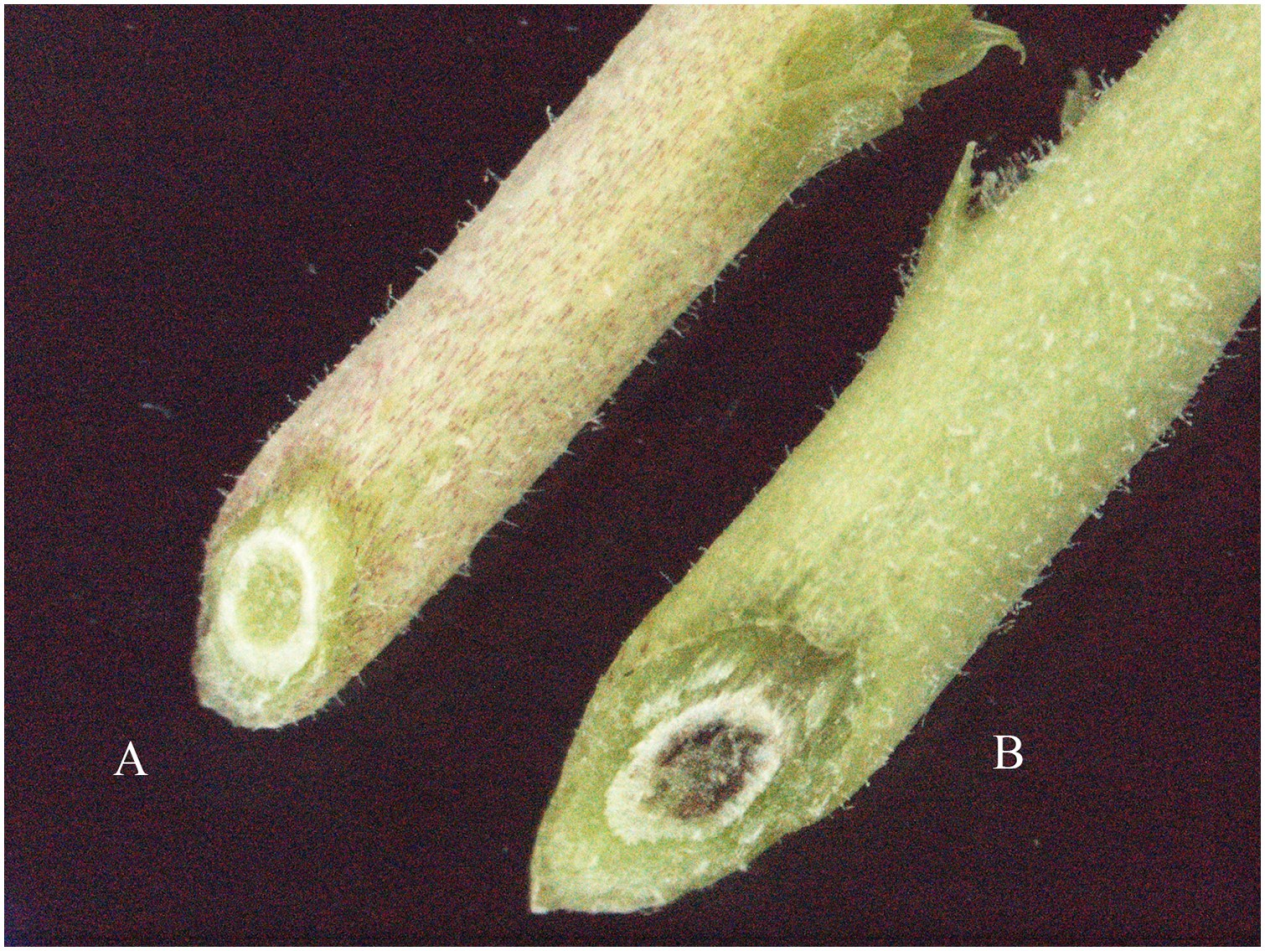
Cross-sectioned stems after six weeks of inoculation. (A) Resistant line to Fusarium race 5 (RIP8-94-5). (B) Susceptible line to Fusarium race 5 (RIP8-94-11).

We considered the experiment suitable for further molecular analysis when the susceptible genotype NIL (RIP8-94-11) and the positive control (Cr5-9) started developing a distinct overall yellow coloration, as compared to the normal appearance in non-inoculated plantlets about two weeks after inoculation with Foc5. Nearly five/six weeks after inoculation, the susceptible plants showed complete wilting, while the resistant inoculated NIL RIP8-94-5 and the non-inoculated plants showed normal healthy growth. Over the course of the experiment, the root length of non-inoculated samples was similar in both, resistant and susceptible lines. On the contrary, related to the inoculated plants, the resistant genotype increased the lateral root development, while the susceptible NIL showed a dark brown, and eventually, dead root system. Cross-sectioned stems of these samples revealed the xylem colonization by the pathogen, while resistant plants showed normal development (Fig 1).

### qPCR assays

Before quantifying the transcript accumulation of the candidate genes, we paid close attention to the preparative stages, which were performed in compliance with the MIQE guidelines [48]. Thus, the main factors of the qPCR workflow that could affect the reliability of the data, such as RNA quality, DNAse treatment, two-step RT-qPCR, use of the same RT master mix that generated one cDNA batch, primer sequences avoiding secondary structures in the amplicon, and PCR efficiency correction, were carefully controlled during the experiment. Additional PCRs were also performed using chickpea genomic DNA as template and gene-specific primers confirming the absence of introns in the amplicon region. Reference genes selection was also a critical step in our analysis and we setup a pilot study to assess the best references to use as internal controls for the broad physiological and cellular changes that are expected to occur during the disease development. The pilot study indicated that *Ca4g26410* and *TFIIA* were the most stable reference genes with stability values lower than the threshold of *M* < 0.5 and CV < 0.25 as defined for acceptable reference genes [40] (S3 Fig). PCR efficiency (E) of the references was respectively: E = 1.987 ± 0.095, E = 1.943 ± 0.071 (mean ± sd). To validate the stable expression of the references in our material, we calculated the mean of their normalized relative quantities. Maximum expression difference between samples for *Ca4g26410* and *TFIIA* was 1.64-fold (S4 Fig).

### Analysis of the interaction time course

We aimed to identify changes in expression levels related to the differential responses of the two NILs (S5 Fig). To best organize our findings, we first show the candidate genes changing in expression levels related to the differential responses against the Fusarium infection. This may be labelled as ‘treatment-dependent regulation’. Next, we show the candidate genes that showed different levels between inoculated plants (resistant vs susceptible NIL; ‘genotype-dependent regulation’). Their potential functions are also further discussed in the second part of this section.

#### Treatment-dependent regulation

**Fig 2:**
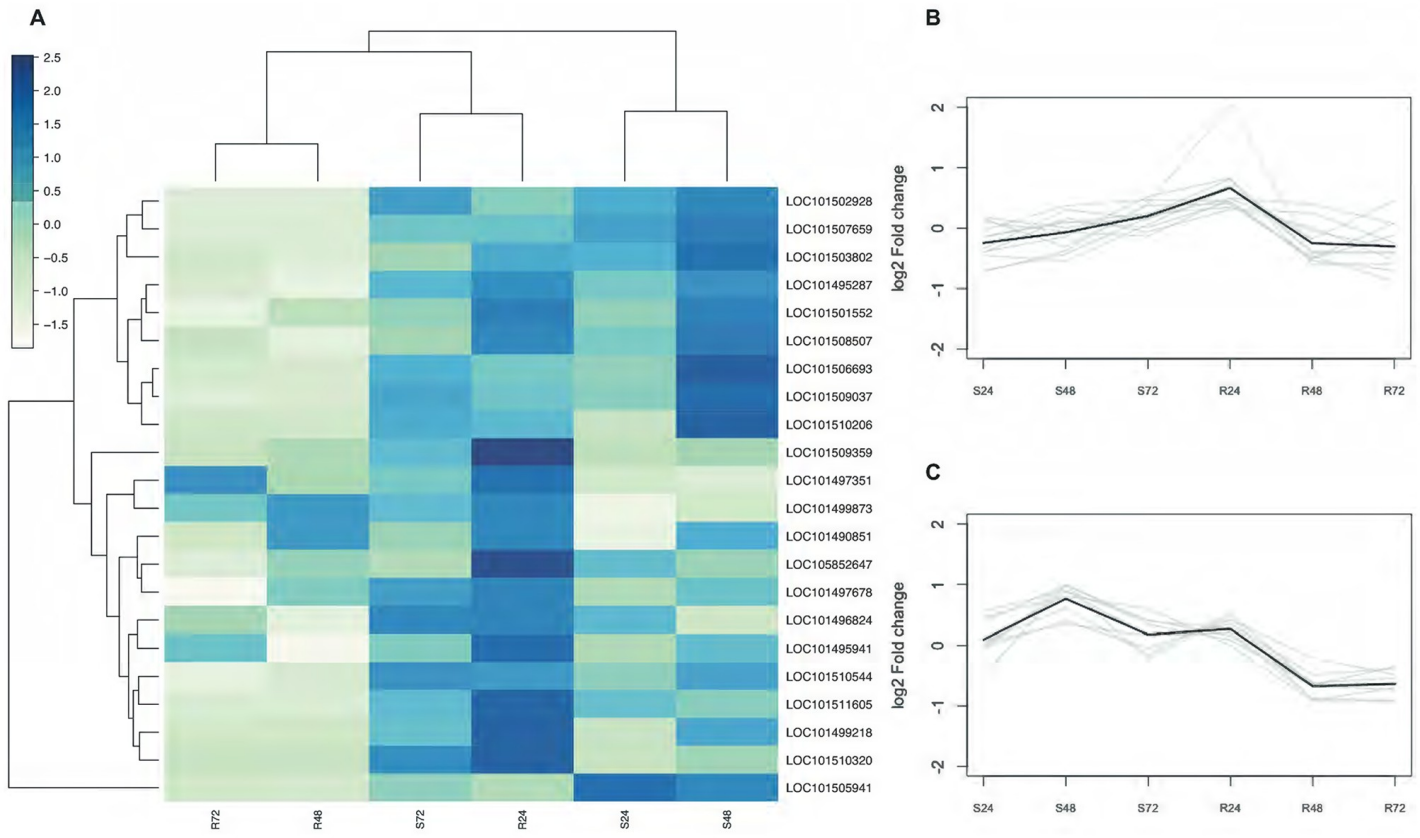
Hierarchical clustering analysis. (A) The heatmap was constructed using the log2-transformed expression levels. Columns represent sample comparisons, while rows represent candidate genes. Colour scale, representing log2 expression differences between inoculated/non-inoculated plants, is shown in the bar. R and S represent resistant and susceptible NILs; 24, 48 and 72 represent hours after inoculation. (B) Expression profiles of differentially expressed genes peaked at 24 hpi in roots of the resistant genotype. (C) Expression profiles of differentially expressed genes peaked at 48 hpi in roots of the susceptible genotype. The mean expression average of each cluster is shown in black.

Hierarchical clustering based on the expression levels of the ratios of the inoculated/non-inoculated plants is presented in Fig 2. Visualizing expression levels through hierarchical clustering gave a sense of the coordinated regulation of genes (Fig 2A). The analysis of their expression levels identified 2 major clusters (one gen remained outside these two clusters). The first cluster grouped 12 genes primarily induced by the resistant genotype at 24 hpi (Fig 2B). The pattern of these samples was similar to the profile exhibited by the susceptible plants at 72 hpi (Fig 2A). According to their GO term-based annotations, their encoded proteins are mostly connected to membranes and their integral components (GO:0016020 and GO:0016021), and associated with signal transduction activities (GO:0007165). The second cluster contained 9 genes mainly induced by the susceptible genotype at 48 hpi (Fig 2C). According to their GO term-based annotations, their proteins are mostly connected to nucleus (GO:0005634) and are involved in the regulation of transcription (GO:0006355).

#### Genotype-dependent regulation

The hierarchical expression profiling analysis revealed not only a differential set of genes expressed under the Foc5 infection, but also a distinct temporal pattern. Thus, we compared the expression profiles of inoculated plants, and found that genes with significantly higher levels in the resistant NIL RIP8-94-5 were mostly activated at 24 hpi, whereas other distinct genes showing higher levels in the susceptible NIL RIP8-94-11 were mostly regulated at 48 hpi. This may suggest that timing of gene regulation is relevant to pathogen recognition, so the host plant can trigger effective defense responses. The two groups of genes are discussed next.

**Fig 3:**
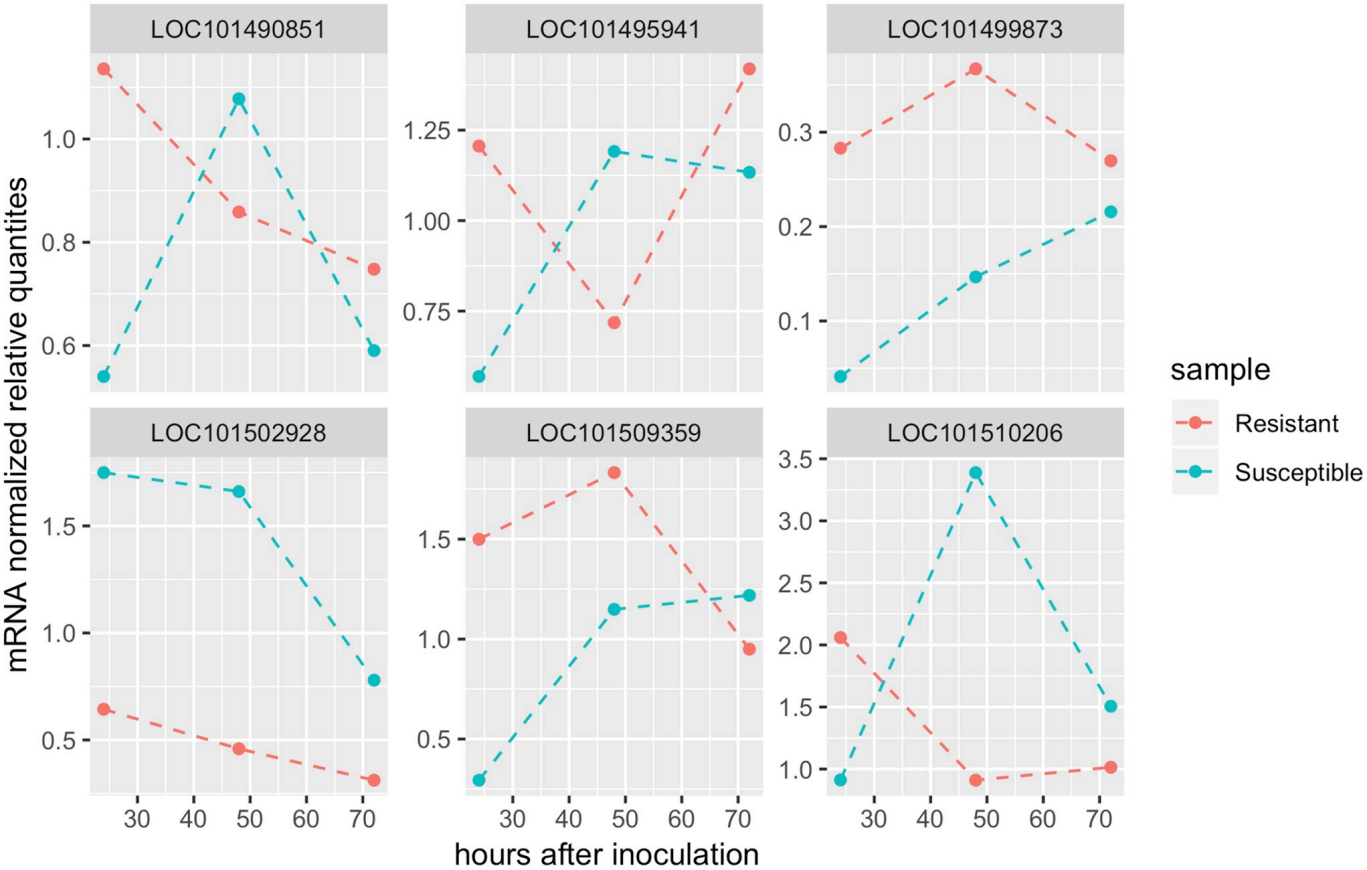
Expression profile of candidate genes in roots of chickpea over the time-course experiment. Red line shows the averaged expression level in resistant NILs, whereas blue line describes the averaged level in the susceptible NILs.

In the first group some genes were found to show higher expression levels in the resistant NIL. The highest expression differences between inoculated plants—resistant vs susceptible—over the experiment were found in the genes LOC101509359 (encoding a MADS-box transcription factor), and LOC101499873 (TMV resistance protein). Both expression differences peaked at 24 hpi (S1 Table). At this time-point, in total 5 genes had higher expression levels in the resistant NIL (Fig 3). Three out of the five genes are located in the genomic region of interest in chromosome 2 [23]. As these loci (LOC101509359, LOC101495941 and LOC101510206) have not been previously linked to defence responses against Fusarium, we labelled them as novel genes. The other two genes have been shown to be induced under Foc1 infection and are located in chromosome 7 and one scaffold non-mapped to the genome [32].

The novel genes, LOC101509359, encodes a MADS-box transcription factor. MADS-box genes are widely described as molecular signatures involved in developmental control and signal transduction in eukaryotes and play many critical regulatory roles [49,50]. In the past decade, a large set of plant MADS transcription factors has been demonstrated to function essentially in diverse biological processes, such as root architecture establishment, gametophyte differentiation, fruit ripening, flowering time regulation, and reproductive organ development [51]. However, stress resistance-related function of MADS-box genes is still not clear [52]. Some MADS-box genes have been found involved in stress-responsive processes beyond growth and development related functions in *Arabidopsis*, Chinese cabbage, wheat, rice, maize and tomato [53–58]. Besides, recent studies highlight that overexpression of a MADS-box transcription factor significantly promoted lateral root development [59,60]. This result is in agreement with the increase of lateral roots that we observed in the resistant inoculated plants.

Another differential expressed novel gene on Ca2 is LOC101495941, which encodes a protein belonging to the multidrug and toxic compound extrusion (MATE) family. MATE genes are involved in various biological activities such as leaf senescence, efflux of antibiotics, aluminium tolerance, vacuole sequestration of plant-derived alkaloids and flavonoids, iron homeostasis and regulation of local auxin biosynthesis [61]. These genes are important as secondary transporters that mediate chemical efflux and play significant roles in salt tolerance in plants [61–64]. In *Arabidopsis*, MATE genes have been shown to be associated with disease resistance [65–67]. Despite some genes in the MATE family have been related to seemingly important physiological functions, most of them are still uncharacterized and the roles of MATE proteins in resistance to pathogens in legumes remains still unknown [68].

The third novel gene on Ca2 is LOC101510206, which encodes for a serine hydroxymethyltransferase. This enzyme is responsible for interconversion of serine and glycine and is essential for cellular one-carbon metabolism providing one-carbon units for a series of important biosynthetic processes such as synthesis of methionine, thymidylates, and purines [69]. *Arabidopsis* mutants defective in serine hydroxymethyltransferase activities have been described more susceptible than control plants to infection with biotrophic and necrotrophic pathogens and have revealed interesting roles in influencing plant defense abilities [70–74]. In legumes, this gene class has been pointed out as responsible for resistance against the nematode *Heterodera glycines* [75]. The expression levels of LOC101510206 is an interesting finding as another locus from the same family mapped onto chromosome 8 has been found to be regulated in chickpea during Foc1 attack [17].

As mentioned above, two genes described previously in the literature as related to defence, LOC101499873 and LOC101490851, showed higher expression levels in the resistant than the susceptible plants at 24 hpi (Fig 3). LOC101499873 is located in a scaffold in the chickpea physical map and described like TMV resistance protein, while LOC101490851, located in chromosome 7, encodes a chaperonin, a molecule class reported to be essential for plants during biotic and abiotic stress conditions [76]. Despite the fact that these two genes are not located within the region of interest controlling the resistance against Foc5 in Ca2, this is an interesting outcome as their transcripts accumulation in early stages of the infection suggests a general network of defence independently of Foc race.

The second group was characterized by a number of genes showing higher expression levels in the susceptible NIL. Nine genes in cluster 2 (LOC101503802, LOC101505941, LOC101506693, LOC101507659, LOC101509037, LOC101510206, LOC101510544, LOC101501552, and LOC101502928; Fig 2C) showed significant higher expression levels in the susceptible NILs at 48 hpi than the resistant at the same time-point. This is the result of transcript induction by susceptible plants, whereas these genes, with the exception of LOC101502928, were not regulated by the resistant. However, LOC101502928, which encodes a WRKY transcription factor, was steadily down-regulated in the resistant over the course of the experiment (S1 Table). WRKY transcription factors may control the defence signalling cascade through a complicated network of genes [77,78]. The downregulation of a defence-related gene is intriguing but that pattern has been already observed in other studies, such as down-regulation of the resistance gene GroES2 in chickpea inoculated with Foc1 [32]. Recently, the silencing of two apricot MATHd genes has been proved to confer resistance against the *Plum pox* virus [79]. There is a need to further explore the exact role of this gene and its interaction with others during defense to understand deeper the regulatory mechanism.

## Concluding remarks

The comparative expression profile between resistant and susceptible inoculated plants showed that both lines are able to sense, and therefore, respond against the fungal attack. However, a number of quantitative and qualitative differences between the genotypes arose during the time-course. The susceptible NIL RIP8-94-11 induces a distinct set of genes that peaked at different time-point and we may speculate whether the response is not strong enough, or expressed at a non-appropriate timing. Conversely, the resistant NIL RIP8-94-5 activates early a defence signalling cascade to protect its primary metabolism from the harmful consequences of pathogenic mayhem, avoiding wilting, and highlighting that proper defence responses during the first hours of fungal attack are crucial to overcome the infection. In this study we have shown that the resistant plants combine an active strategy of defence against Foc5 by up-regulating the expression of three genes (LOC101509359, LOC101495941 and LOC101510206) with a general defensive line independent of Foc race.

NILs, like those used in this study, have the advantage that only a small target region of the genome is segregating, consequently, the genetic background noise is uniform. Different expression patterns could be considered more reliable than other plant material. On the basis of this consideration future re-sequences of this pair of NILs could give us the opportunity to find key differences in their genomes.

## Supporting information

S1 Fig Modeling of secondary structures of the amplicons for the assays designed in this study. Thermodynamic stability (ΔG, kcal/mol) is presented in the figure. Primers are indicated by black arrows. Although some secondary structures might be present where primers anneal for some assays, they have a positive ΔG value and Tm < 60 °C, and hence will not influence the amplification efficiency.
(PDF)

S2 Fig Dissociation curves for the 22 PCR products. (PDF)

S3 Fig Stability ranking of four reference genes from chickpea series. Numbers on top of the bars indicate the CV values of the reference involved in the normalization. References showing the highest stable expression (*M* < 0.5 and CV < 0.25) are represented in black colour.
(TIFF)

S4 Fig Evaluation of reference genes for chickpea samples. (TIFF)

S5 Fig Alluvial diagram showing regulation of candidate genes over the time-course experiment in A) resistant, and B) susceptible plants. (PDF)

S1 Table Expression ratios of genes in resistant and susceptible plants over the time-course experiment. Values indicate log2 average expression ratios of infected plants (resistant / susceptible). Bold text indicates statistically significant regulation (P < 0.05).
(DOCX)
