# Supplementary figures and images for: Candidate genes expression profiling during wilting in chickpea caused by Fusarium oxysporum f. sp. ciceris race 5
Source: PLoS One. 2019 Oct 23;14(10):e0224212. doi: 10.1371/journal.pone.0224212 (PMC6808423; doi:10.1371/journal.pone.0224212)

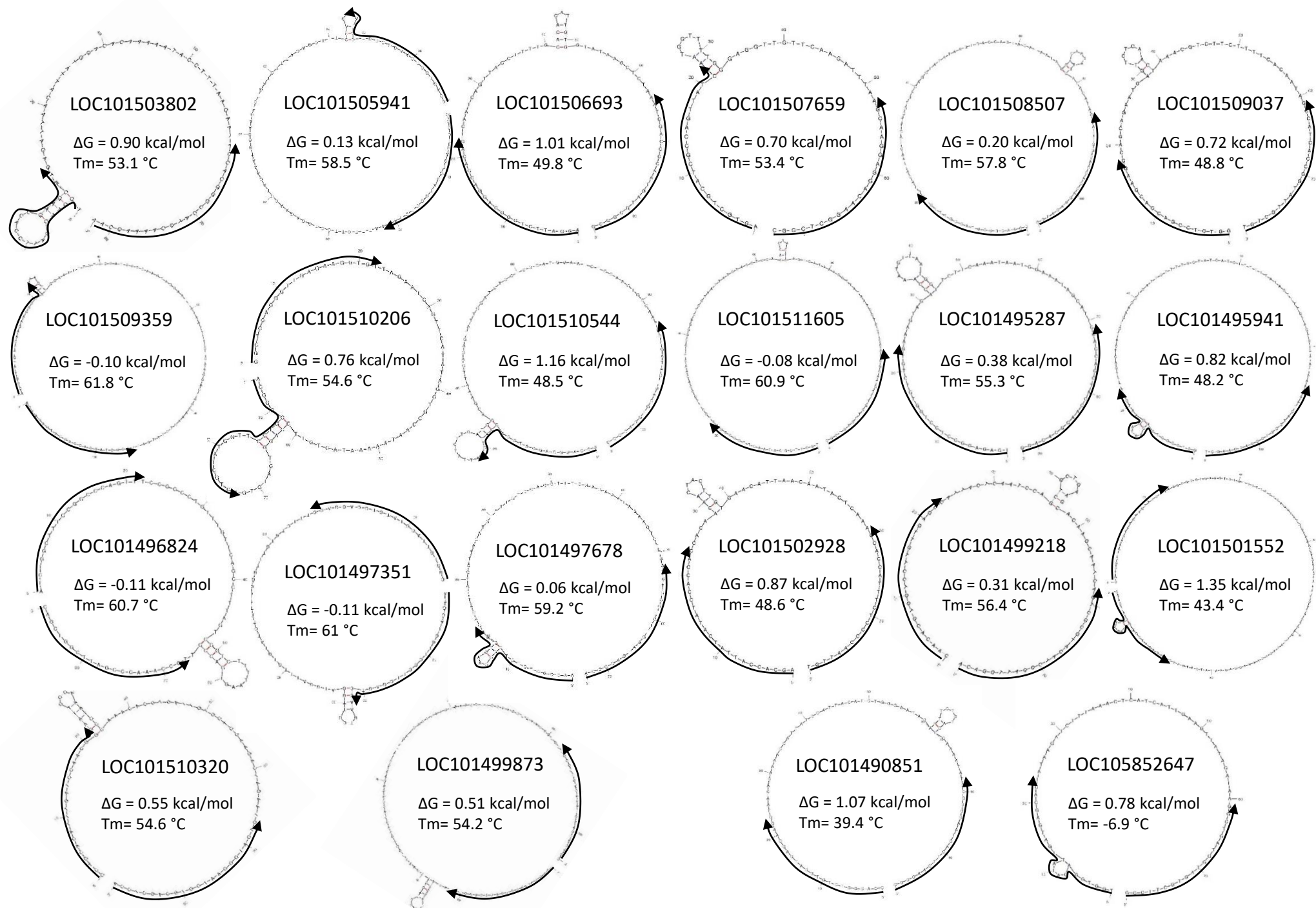

Supplement: S1 Fig — Thermodynamic stability (ΔG, kcal/mol) is presented in the figure. Primers are indicated by black arrows. Although some secondary structures might be present where primers anneal for some assays, they have a positive ΔG value and Tm < 60 °C, and hence will not influence the amplification efficiency. (PDF) [file pone.0224212.s001.pdf]

LOC101503802

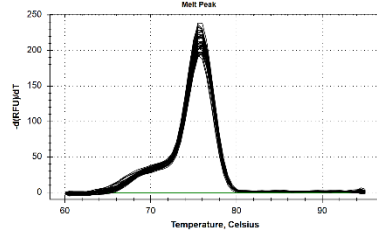

LOC101505941

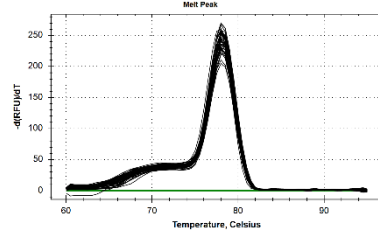

LOC101506693

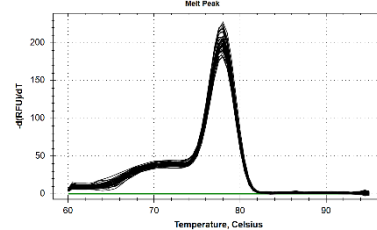

LOC101507659

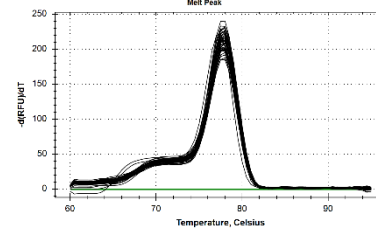

LOC101508507

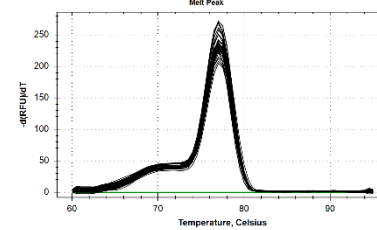

LOC101509037

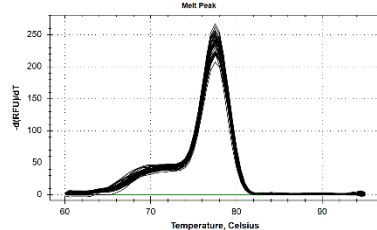

LOC101509359

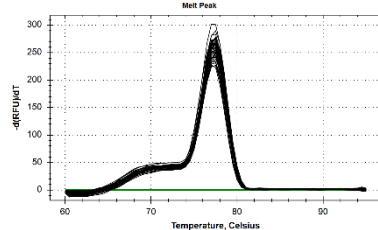

LOC101510206

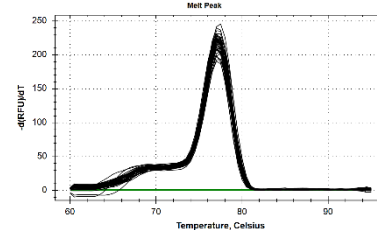

LOC101510544

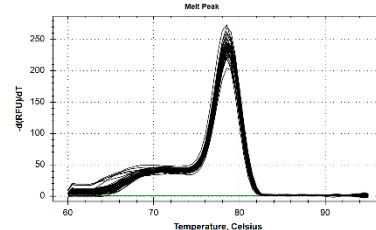

LOC101511605

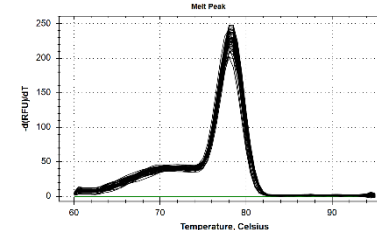

LOC101495287

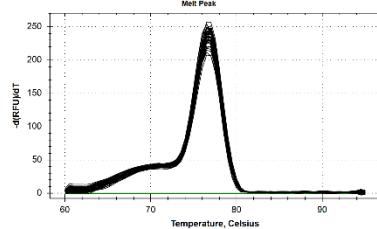

LOC101495941

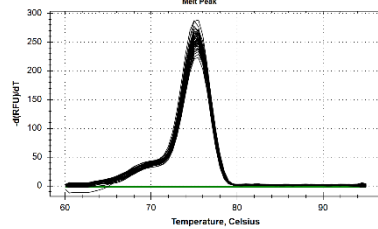

LOC101496824

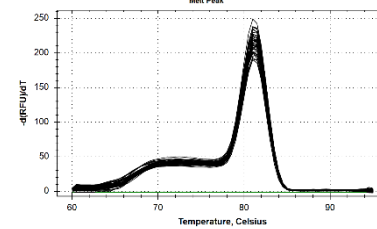

LOC101497351

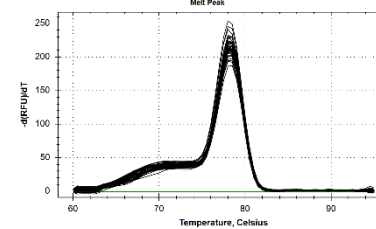

LOC101497678

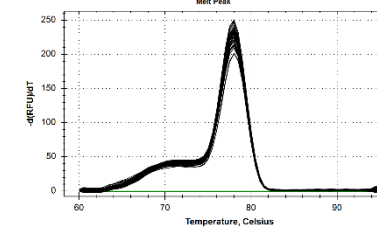

LOC101502928

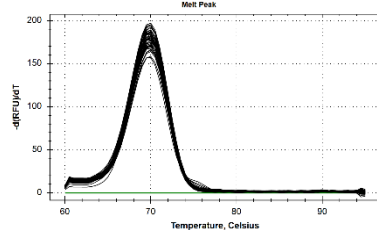

LOC101499218

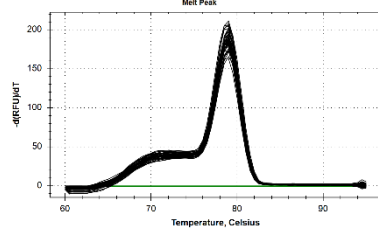

LOC101501552

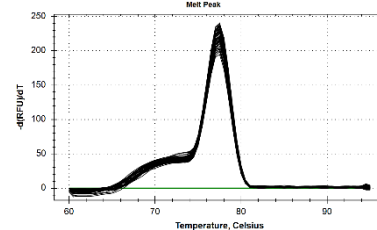

LOC101510320

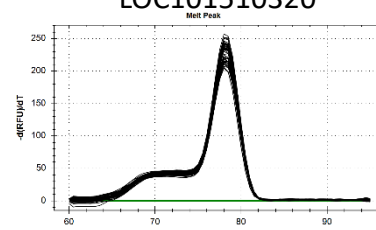

LOC101499873

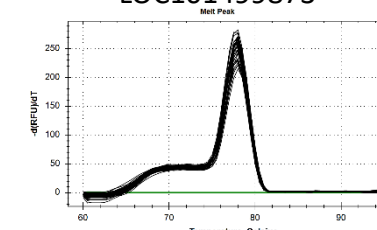

LOC101490851

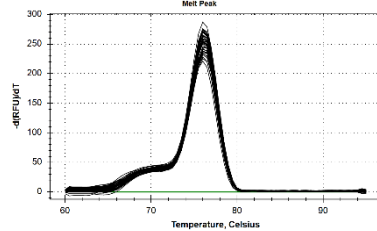

LOC105852647

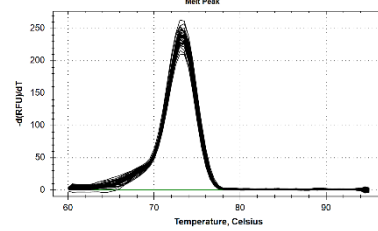

Supplement: S2 Fig — (PDF) [file pone.0224212.s002.pdf]

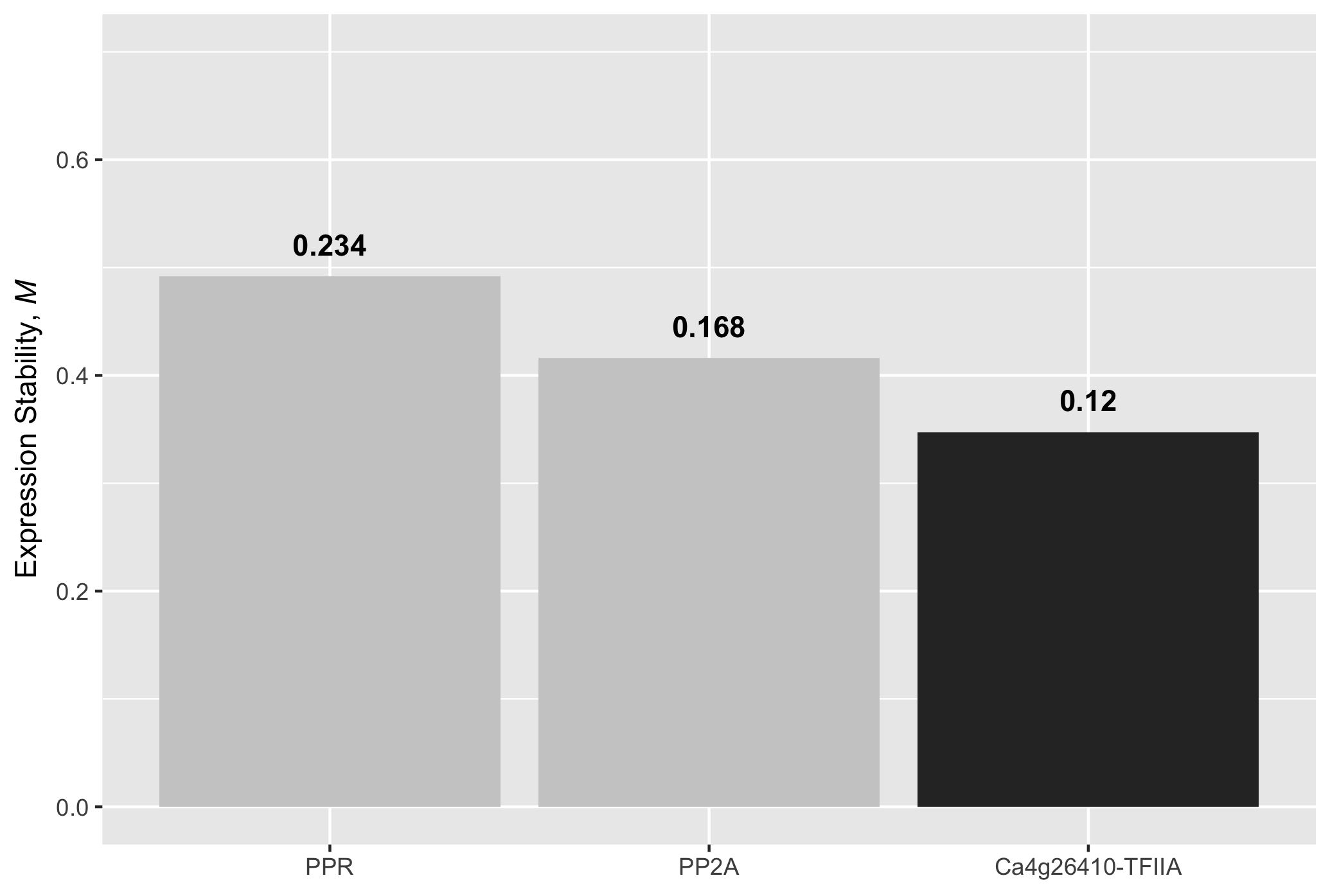

Supplement: S3 Fig — Numbers on top of the bars indicate the CV values of the reference involved in the normalization. References showing the highest stable expression (M < 0.5 and CV < 0.25) are represented in black colour. (TIFF) [file pone.0224212.s003.tiff]

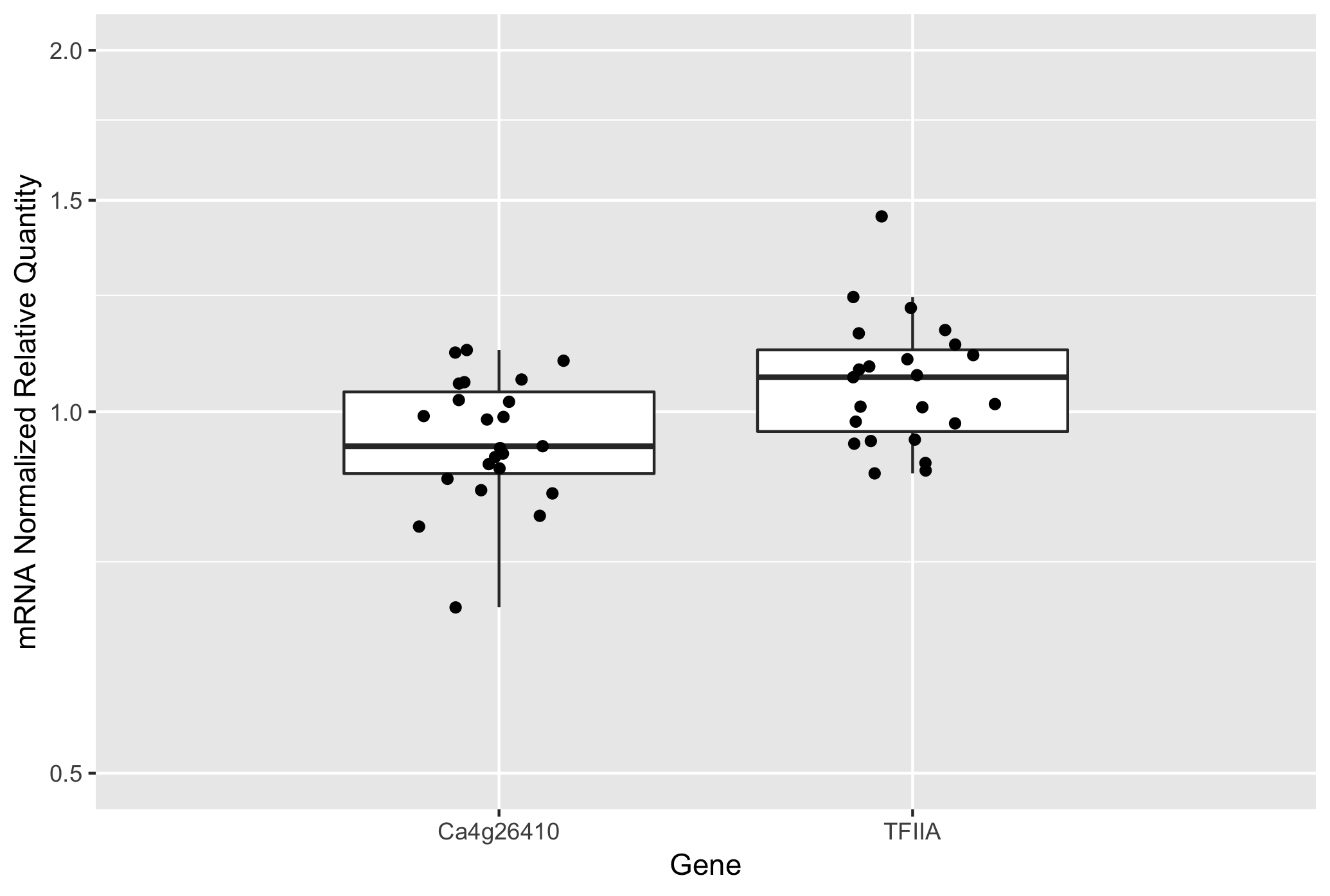

Supplement: S4 Fig — (TIFF) [file pone.0224212.s004.tiff]
