# Supplementary material for: Candidate genes expression profiling during wilting in chickpea caused by Fusarium oxysporum f. sp. ciceris race 5
Source: PLoS One. 2019 Oct 23;14(10):e0224212. doi: 10.1371/journal.pone.0224212 (PMC6808423; doi:10.1371/journal.pone.0224212)
Supplement: S1 Table — Values indicate log2 average expression ratios of infected plants (resistant / susceptible). Bold text indicates statistically significant regulation (P < 0.05). (DOCX) [file pone.0224212.s006.docx]

**S1 Table. Expression ratios of genes in resistant and susceptible plants over the time-course experiment.** Values indicate log2 average expression ratios of infected plants (resistant / susceptible). Bold text indicates statistically significant regulation ( P < 0.05).

| **Gene ID** | **24 hpi** | **48 hpi** | **72 hpi** |
| --- | --- | --- | --- |
| LOC101503802 | 0.21 | **-1.54** | -0.58 |
| LOC101505941 | -0.52 | **-1.98** | **-1.02** |
| LOC101506693 | 0.43 | **-1.54** | -0.53 |
| LOC101507659 | -0.21 | **-1.60** | **-1.16** |
| LOC101508507 | 0.83 | -0.60 | 0.30 |
| LOC101509037 | 0.62 | **-1.53** | -0.89 |
| LOC101509359 | **2.35** | 0.67 | -0.36 |
| LOC101510206 | **1.18** | **-1.90** | -0.57 |
| LOC101510544 | 0.51 | **-1.17** | **-1.31** |
| LOC101511605 | 0.40 | -0.60 | -0.78 |
| LOC101495287 | 0.70 | -0.78 | -0.46 |
| LOC101495941 | **1.08** | -0.73 | 0.32 |
| LOC101496824 | 0.36 | 0.34 | -0.44 |
| LOC101497351 | 0.84 | 0.55 | 0.56 |
| LOC101497678 | 0.44 | -0.41 | -0.48 |
| LOC101502928 | **-1.44** | **-1.86** | **-1.32** |
| LOC101499218 | 0.63 | -0.51 | -0.45 |
| LOC101501552 | 0.10 | **-1.13** | -0.36 |
| LOC101510320 | 0.39 | -0.29 | -0.94 |
| LOC101499873 | **2.78** | 1.32 | 0.32 |
| LOC101490851 | **1.07** | -0.33 | 0.34 |
| LOC105852647 | 0.58 | -0.08 | -0.03 |
