# Supplementary material for: Candidate genes expression profiling during wilting in chickpea caused by Fusarium oxysporum f. sp. ciceris race 5
Source: PLoS One. 2019 Oct 23;14(10):e0224212. doi: 10.1371/journal.pone.0224212 (PMC6808423; doi:10.1371/journal.pone.0224212)
Supplement: S5 Fig — (PDF) [file pone.0224212.s005.pdf]

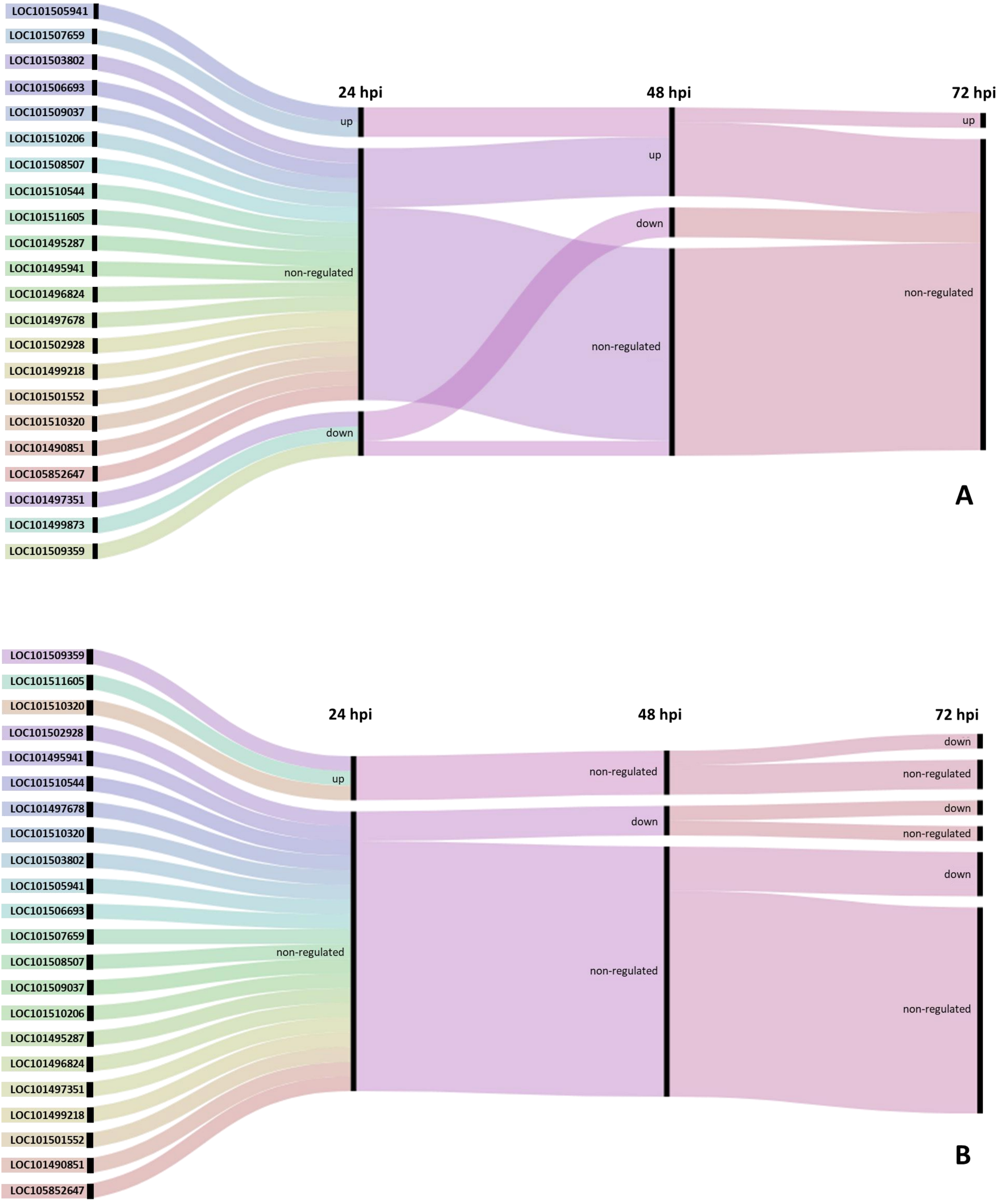
LOC101505941
LOC101507659
LOC101503802
LOC101506693
LOC101509037
LOC101510206
LOC101508507
LOC101510544
LOC101511605
LOC101495287
LOC101495941
LOC101496824
LOC101497678
LOC101502928
LOC101499218
LOC101501552
LOC101510320
LOC101490851
LOC105852647
LOC101497351
LOC101499873
LOC101509359
24 hpi
48 hpi
72 hpi
up
non-regulated
down
up
down
non-regulated
up
non-regulated
A
LOC101509359
LOC101511605
LOC101510320
LOC101502928
LOC101495941
LOC101510544
LOC101497678
LOC101510320
LOC101503802
LOC101505941
LOC101506693
LOC101507659
LOC101508507
LOC101509037
LOC101510206
LOC101495287
LOC101496824
LOC101497351
LOC101499218
LOC101501552
LOC101490851
LOC105852647
24 hpi
48 hpi
72 hpi
up
non-regulated
non-regulated
down
non-regulated
down
non-regulated
down
non-regulated
down
non-regulated
B
